# Supplementary material for: Expectation-Maximization Binary Clustering for Behavioural Annotation
Source: PLoS One. 2016 Mar 22;11(3):e0151984. doi: 10.1371/journal.pone.0151984 (PMC4803255; doi:10.1371/journal.pone.0151984)
Supplement: S1 Text — (PDF) [file pone.0151984.s001.pdf]

Supporting Information
Expectation-Maximization Binary Clustering for Behavioural Annotation

Joan Garriga, John R.B. Palmer, Aitana Oltra, Frederic Bartumeus

A Movement Variables

We focus our analysis on two elementary movement behaviour variables: velocity and turning angles. Note, however, that the framework is multivariate and extendable to any type of biologging behavioural variables (e.g. heart rate beat, accelerometry) and/or environmental variables (e.g. wind, landcover).

To compute velocity and turns, we first calculate distances and directions between successive pairs of locations. Following EURING (www.euring.org) recommendations and to avoid spatial distortions associated to projected coordinate systems (especially important at global scales, e.g. migration), we calculate distances as loxodromic lines using the following expressions (Figure A):

Given two points $(lat_i, lon_i), (lat_j, lon_j)$,

1. we define,

$$\Delta\varphi = ln\left(\frac{tan\left(\frac{lat_j}{2}+\frac{\pi}{4}\right)}{tan\left(\frac{lat_i}{2}+\frac{\pi}{4}\right)}\right) \tag{1}$$

   where $\Delta\varphi$ is the stretched difference between latitudes lat_i, lat_j in radians;

2. assuming coordinates are in decimal degrees, the differences in latitude and longitude expressed in radians are,

$$\Delta lon = (lon_j - lon_i)\frac{\pi}{180}$$
$$\Delta lat = (lat_j - lat_i)\frac{\pi}{180}$$

3. by use of equation 1, we define,

$$\text{if } \Delta lat = 0\,, \quad q = cos(lat_i) \tag{2}$$
$$\text{else }, \quad q = \frac{\Delta lat}{\Delta\varphi} \tag{3}$$

   where q is the latitude correction factor to adjust Δlon.

A.1 Distances

Given the above expressions, distances are calculated by the Pythagorean Theorem,

$$d_{ij} = R\sqrt{\Delta lat^2 + (q\Delta lon)^2}$$

where $R = 6378160.0$ is the mean earth radius in meters.

A.2 Absolute Directions

The usual practice is to indicate directions in degrees from North clockwise. Therefore, we use the following procedure,

1. first we consider the special cases,

$$\begin{aligned}
\text{if } \Delta lon = 0 \text{ and } \Delta lat = 0\,, &\quad \theta_i = 2\pi \quad \text{(no move)}\\
\text{elif } \Delta lon = 0 \text{ and } \Delta lat > 0\,, &\quad \theta_i = 0 \quad \text{(moving north)}\\
\text{elif } \Delta lon = 0 \text{ and } \Delta lat < 0\,, &\quad \theta_i = \pi \quad \text{(moving south)}\\
\text{elif } \Delta lon > 0 \text{ and } \Delta lat = 0\,, &\quad \theta_i = \frac{\pi}{2} \quad \text{(moving east)}\\
\text{elif } \Delta lon < 0 \text{ and } \Delta lat = 0\,, &\quad \theta_i = \frac{3\pi}{2} \quad \text{(moving west)}
\end{aligned}$$

   (note that if two successive locations lie exactly over the same coordinates, we use 2π to differentiate this case from moving north);

2. by use of Eq. 3 we get,

$$\begin{aligned}
\text{if } \Delta lon = 0\,, &\quad tan\,\bar{\theta}_i = 0\\
\text{else }, &\quad tan\,\bar{\theta}_i = \frac{\Delta lat}{q\,\Delta lon} = \frac{\Delta\varphi}{\Delta lon},
\end{aligned}$$

   where $\bar{\theta} = \left(\frac{\pi}{2} - \theta_i\right)$

3. values of $\bar{\theta}_i$ are given by,

$$\begin{aligned}
\text{if } \Delta lon > 0\,, &\quad \bar{\theta}_i = atan2\left(tan\,\bar{\theta}_i, 1\right)\\
\text{else }, &\quad \bar{\theta}_i = atan2\left(-tan\,\bar{\theta}_i, -1\right)
\end{aligned}$$

4. *atan2* yields values in the range $0 < \bar{\theta}_i < \pi$ (anticlockwise) and $-\pi < \bar{\theta}_i < 0$ (clockwise) with respect to the horizontal axis, so we can refer them to north clockwise with,

$$\begin{aligned}
\text{if } \Delta lat > 0\,, &\quad \theta_i = fmod\left(\frac{5\pi}{2} - \bar{\theta}_i, 2\pi\right)\\
\text{else }, &\quad \theta_i = \frac{\pi}{2} - \bar{\theta}_i
\end{aligned}$$

A.3 Velocity

Although animals are sometimes equipped with special devices to register instantaneous velocity, we refer here to the more general case of estimating velocities by computing differences in successive locations. This computation can lead to unreliable information when time gaps in between locations are too long, but it is at this point when the implementation of uncertainty in the EMbC comes clearly useful.

Generally, given two successive points velocity is computed as,

$$v_i = \frac{d_{i,i+1}}{t_{i+1} - t_i}. \tag{4}$$

If projected coordinates are provided, then, given $(t_i, x_i, y_i)\,, (t_{i+1}, x_{i+1}, y_{i+1})$, we can alternatively calculate velocity as,

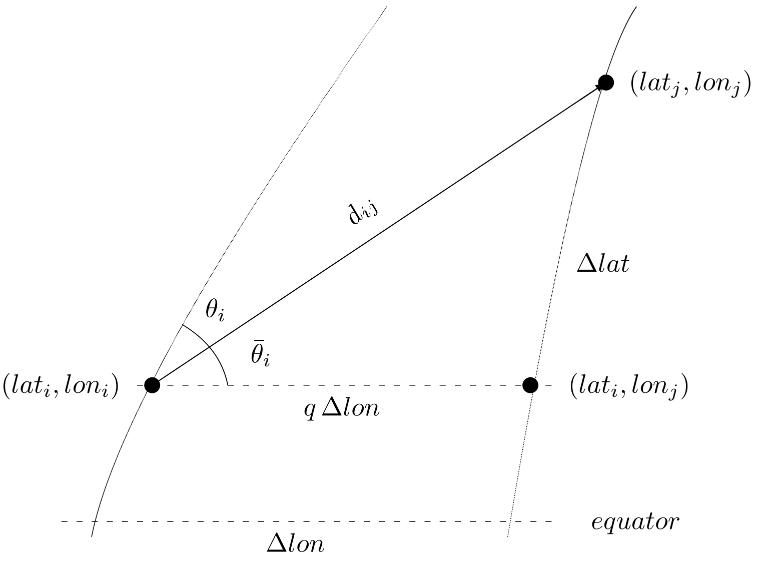

Figure A: Correction of Δlon for the computation of the loxodromic distance and direction.

$$v_i = \frac{\sqrt{(x_{i+1} - x_i)^2 + (y_{i+1} - y_i)^2}}{t_{i+1} - t_i} \tag{5}$$

Note that the *velocity* assigned to any location is calculated with respect to the next location, not the previous one. This is important in order to correctly assign velocities to the limiting points of a period of relocation.

A.4 Turn

The variable *turn*, denoted by α_i, is defined as the absolute value of the difference between the *absolute direction* at location i with respect to the previous direction, that is,

$$\alpha_i = min\left[abs\left(\theta_i - \theta_{i-1}\right), \left(2\pi - abs\left(\theta_i - \theta_{i-1}\right)\right)\right]. \tag{6}$$

Note that turns are never considered to be greater than π.

While θ_i (absolute direction at location i) is forwardly calculated, α_i (*turn* at location i) is calculated with respect to the previous direction.

A special case is when two successive points lie on the same coordinates and it is therefore impossible to determine an absolute direction. As explained in Section A.2, we mark up this situation by $\theta_i = 2\pi$, and we assume that the animal was resting in the meantime. Consequently, whenever we have either $\theta_i = 2\pi$ or $\theta_{i-1} = 2\pi$, we force $\alpha_i = 0$. The latter expresses the idea that, after a period of resting, there is an absolute direction of departure but it makes no sense to compute a turn value. This convention is useful for the distinction of resting and foraging behaviours when velocity values are low.

B *Caenorhabditis elegans* movement analysis

The *C.elegans* dataset consists of video-records of the centre of mass of 6 worms (32Hz of resolution for 90 min period of observation) on a temperature controlled plate (24.5 × 24.5 cm) at approximately 21C.

Cluster	Straightness	Mean velocity	Net displacement
LLL	1.7272687	0.2141672	3.0844566
LLH	4.2097544	0.3064891	7.0157562
LHL	3.3697537	0.4547349	4.8050367
LHH	6.1634326	0.4975237	16.8821735
HLL	6.1966796	0.4161725	11.5659529
HLH	9.734585	0.430205	30.463736
HHL	9.4242524	0.5869336	14.1155785
HHH	10.8363510	0.4959341	34.3040610

Table A: ***C.elegans* clustering results**. Mean values of the three input features in each output cluster.

All worms were cultivated under the same temperature conditions as the assay. Individuals were rinsed of *E.coli* by transferring them from OP50 food plates into M9 buffer (same inorganic ion concentration as M9 assay plates) and letting them swim for 1 min. Individual worms were transferred from the M9 buffer to the centre of the assay plate. First 5 minutes were not tracked.

In high resolution trajectories, any movement variable computed by means of locally defined spatial measures present two problems:

1. measures corresponding to successive locations are highly correlated;
2. measures at each single location are extremely local.

This impairs the possibility to capture large-scale movement structures. Therefore, our first step is to reduce the correlation by undersampling the trajectories at a given fix rate (0.33 Hz). Secondly, in order to avoid extremely local measurements, we introduce some spatial measures based on averaged values within a given time window (5 minutes) which defines a set of neighbouring locations.

Let's denote by N_i the neighbourhood of any location i in the trajectory, given as a subset of successive locations centred on i. Let's denote by l, r the leftmost and rightmost locations in any neighbourhood set. Let's also denote by d_{ij} the euclidean distance between any two locations i, j.

Given the above, we define three spatial measures. All of them are similar in order of magnitude and smoothing scope. Note that the values and final clustering semantics depend on the neighbourhood size, however, in the definitions below we have tried to avoid as much as possible this dependency by normalizing by the number of neighbours.

- **Straightness Index**

$$C_i = \frac{2}{|N_i|} \sum_{j \in N_i} d_{ij} \tag{7}$$

This is an inverse measure of the spatial aggregation of neighbouring locations, intended to convey information about the intensity of local search (although inverse).

- **Net displacement**

$$R_i = \frac{1}{|N_i|} \sum_{j \in N_i} d_{l,r}^{(j)} \tag{8}$$

This is equivalent to a mean *net displacement* over all locations in the neighbourhood of i, where $d_{l,r}^{(j)}$ refers to the net displacement for each location $j \in N_i$ including i itself. This one is intended to convey information about the will of the individual to move towards a different location.

- **Mean velocity**

$$T_i = \frac{\bar{N}}{|N_i|} \sum_{j \in N_i} d_{j,j+1} \tag{9}$$

This is equivalent to a mean *gross displacement* measure or mean *travelled distance*, where $\bar{N}$ is the mean neighbourhood size and scales the measure to orders of magnitude similar to the other measures defined. This measure is intended to convey information about the velocity in the movement of the individual.

C Data reliability functions

Similarly to [1, 2], the reliability of the data is implemented as an additional weighting coefficient in Equations 3 and 4 in the main text, giving less weight to the less accurate values in the estimation of the Gaussian parameters, and favouring the more accurate ones (Equations 6 and 7 in the main text). These coefficients should be given by a *reliability* function that can not be generalized, as it will be variable-specific and dependent on the source of error considered. As a general rule, however, it should yield normalized reliability values.

An adequate *reliability* function to account for the uncertainty in the velocity values due to heterogeneous sampling rates, can be defined in terms of the time interval $\tau_i = t_{i+1} - t_i$ associated to location i with timestamp t_i,

$$u_i^{(l)} = min\left(\frac{\hat{\tau}}{\tau_i}, 1\right) \tag{10}$$

where the superscript l makes reference to the vector index of the variable velocity, and $\hat{\tau}$ is the most frequent time interval (the mode of the τ frequency distribution), for which we assume the baseline of reliability of the trajectory, so that for $\tau_i \leq \hat{\tau}$ we assign a maximum reliability $u_i^{(l)} = 1$, with $u_i^{(l)}$ decreasing as τ_i increases.

Equation 10 can be generalized to q uncertainty components to include other sources of error leveraging on the measured variable (*e.g.* the spatial error of the sampling device). In this case we consider a vectorial reliability function $\boldsymbol{u}_i^{(l)} = (u_i^{(l,1)}, \ldots, u_i^{(l,q)})$ and take its normalized length as given by,

$$u_i^{(l)} = \frac{1}{\sqrt{q}} \left[\sum_{c=1}^{q} \left(u_i^{(l,c)}\right)^2\right]^{1/2} \tag{11}$$

D Bursted visualization of annotated trajectories

A relevant aspect of movement behaviour annotation is finding adequate ways to visualize the results. We propose a visualization based on three dimensions: space, time, and behaviour, using coloured lines and circles (colour for behaviours, line lengths for space, and circle radius for time). We use this type of visualization in Figures 6 and 7 in the main text. This involves the conversion into unique lines of all consecutive locations sharing the same label (*bursted* visualization). At the mid-point of each generated burst we associate a circle with radius proportional to the duration of the behaviour. This enables the viewer to discern the distance travelled and the duration of each behaviour simultaneously. If distance travelled is small but duration is large, the circle dominates the line. If the opposite is true, the line dominates over the circle.

E EMbC likelihood dynamics

As implemented in the EMbC, the step-wise computation of the Gaussian parameters (Equations 6 and 7 in the main text) does not result in a strict maximization of the likelihood. This is in fact a pseudo-maximization step expressing a trade-off between two distinct objective functions driving the algorithm: finding a binary partition, and finding a maximum likelihood partition, with preference for the first one. Therefore, any discrepancy arising between them will result in a drop in likelihood. Assuming our interest in a binary partition even at some cost in likelihood, such a pseudo-maximization gives rise to the following important features (illustrated in Figure B): (i) the algorithm avoids getting stuck in local maximums that do not correspond to a stable binary partition, (ii) a cluster can vanish after being absorbed by adjacent clusters (a vanishing cluster is usually associated to a decrease in likelihood but the likelihood can increase again later on), and (iii) the output does not always correspond to a strict four quadrant layout.

In Figure B we compare the likelihood dynamics and the clustering resulting from the EMC and the EMbC algorithms. The aim is to depict the effect of this trade-off between the maximum likelihood and the binary partition, simplifying the clustering semantics. Each column in Figure B is an example of a synthetically generated dataset based on a GMM with four Gaussian components. The GMMs were generated by randomly sampling the set of parameters $\Theta = \{\boldsymbol{\mu}_j, \boldsymbol{\Sigma}_j, \pi_j\}$, $1 \leq j \leq 4$, following the protocol described in Section F. Afterwards, a random dataset of size $n = 2000$ was drawn from each GMM.

While the EMC yields apparently better results, both in terms of clustering (*i.e.* better match of the four clusters) and likelihood, the distinction of some of the clusters appears merely as a result of forcing four clusters and it can hardly be translated into a clear semantics. Note that, as these are synthetic datasets, we could inform the EMC with the correct number of clusters but this is not the case in any unsupervised empirical problem. In contrast, the likelihood pseudo-maximization implemented in the EMbC, allows the algorithm to proceed through some steps of decreasing likelihood and find its way to alternative stable solutions, overcoming a drop in likelihood due to a vanishing cluster (left example), or following a long way of decreasing steps to end up merging two clusters (centre example). In the case of an underlying GMM with largely overlapped Gaussian components (right example), the EMbC, which is not constrained by the assumption of four clusters, can yield a more realistic partition, although after a long and steady decrease in likelihood. In general, the clustering layout of the EMbC yields simpler semantics than the EMC in terms of high/low values of the input features.

F Generation of synthetic trajectories

Our aim in using synthetic trajectories was not only to numerically assess the performance of the EMbC algorithm, but also to compare it with related algorithms like the EMC and the HMM to clearly depict the gap the EMbC fills among these algorithms. Therefore, we generated a set of synthetic trajectories representing the use case of the EMbC, *i.e.* under the hypothesis of an underlying bivariate GMM with four components representing the four binary regions of a bivariate binary clustering (LL, LH, HL and HH).

In order to cover the broadest variability in the shape of underlying GMMs, while somehow preserving a match of the Gaussian components with a binary split of the bivariate (speed/turn) space, we generated GMMs by randomly sampling the set of parameters $\Theta = \{\boldsymbol{\mu}_j, \boldsymbol{\Sigma}_j, \pi_j\}$, $1 \leq j \leq 4$ as follows:

- the set of prior probabilities π_j was randomly drawn from a Uniform distribution bounded to the range $0.25 \leq \pi_j \leq 0.75$ to assure a minimum balancing in the representation of the four clusters.
- the set of means $\boldsymbol{\mu}_j = (\mu_{speed}, \mu_{turn})_j$ was randomly drawn from a Beta(1,6) distribution for low values and a Beta(6,1) for high values. The sampled values, lying in the interval $(0, 1)$, were

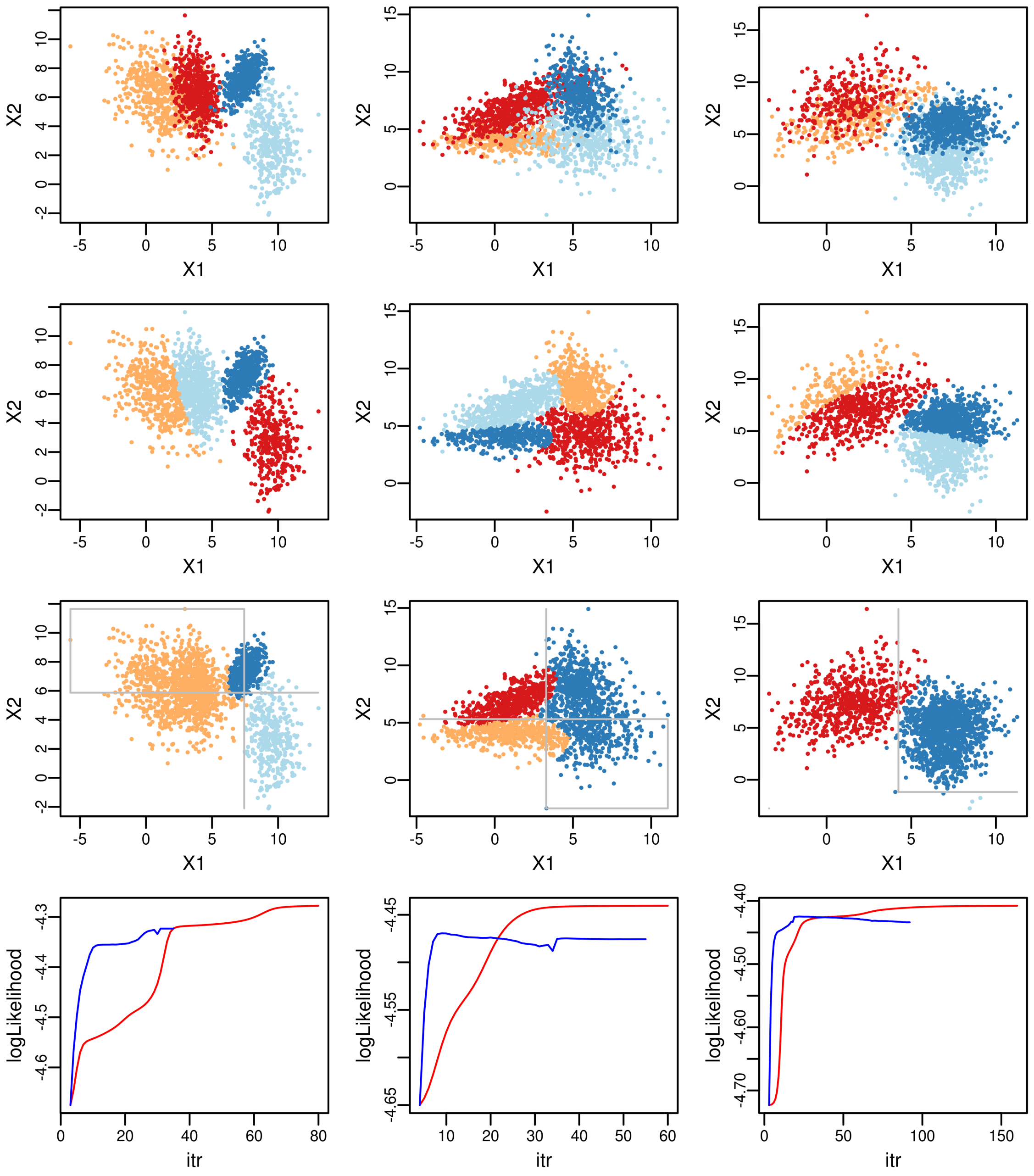

Figure B: **Likelihood dynamics** Each column shows an example of a synthetically generated dataset based on a GMM with four Gaussian components. Row 1: scatter plot of the sampled data, with data points coloured accordingly to the generative Gaussian component; Row 2: scatter plot of the output clustering of the EMC (note that the EMC does not preserve the reference colour labelling in Row 1); Row 3: scatter plot of the binary clustering of the EMbC (with same colour coding as in Row 1) and grey lines depicting the values of the delimiters; Row 4: plots showing the corresponding likelihood dynamics, EMC (red) and EMbC (blue).

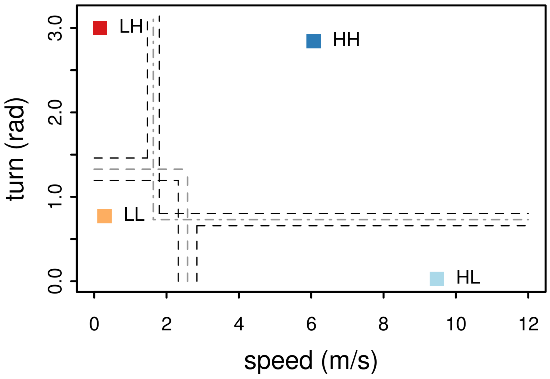

Figure C: **Generation of synthetic GMMs and the corresponding binary regions.** The set $\Theta = \{\boldsymbol{\mu}_j, \boldsymbol{\Sigma}_j, \pi_j\}$, $1 \leq j \leq 4$ of GMM parameters is randomly sampled. In order to get more realistic partitions and consequently more realistic paths, the delimiters are initially set to a fourth of the distance between the means (coloured squares), as measured from the low mean, ($r_{.L}$, $r_{L.}$ in grey dashed lines, and $r_{.H}$, $r_{H.}$ in grey dot-dashed lines). The γ parameter defines the width of the boundary around the delimiters (black dashed lines), that further delimit the binary regions. Data points are sampled from truncated Gaussians based on Θ and limited to the region boundaries.

afterwards spanned to the range $0 \leq \mu_{speed} \leq 10$ and $0 \leq \mu_{turn} \leq \pi$.

- the covariance matrices Σ_j were build by drawing values from a Beta(2,2) distribution compounded with a bounded Uniform(1,2) distribution for variances and a bounded Uniform(-4,0) for covariances, spanning the values of the variances as above.

We also wanted to have some control over the definition of the resulting clusters. To this aim the delimiters were set at a midpoint between the means of the four Gaussian components. Afterwards, we forced a boundary of forbidden values around the delimiters by means of a parameter $\gamma = \{0.01, 0.05, 0.1\}$ given as a fraction of the distance between the means (see Figure C). Note that γ does not determine equal widths for all boundaries nor prevents potential overlapping of the binary regions thus contributing further randomness in the generation of our trajectories set.

The next step was to sample the sequence of states of the trajectory (the expected labels of the resulting clusters), for which we used two different sampling schemes:

1. a Markov-chain based sampling scheme, that is, we randomly sample an extra set of parameters from a beta distribution (a different one for each state) to define a multinomial transition matrix, sampling state labels based on that transition matrix;

2. a mixture prior based sampling scheme, that is, state labels were sampled based only on the prior parameters π_j, $1 \leq j \leq 4$.

Following the sequence of states, velocity/turn pairs were sampled from truncated Gaussians corresponding with the limits of each binary region using a Gibbs sampling algorithm. Also, a sequence of time intervals was randomly sampled from a $\mathcal{N}(1,1) * 1800$ seconds, *i.e.* setting trajectory locations at a mean time interval of half an hour.

Finally, given a starting location, the trajectories were generated computing the geolocations derived from the velocity/turn values and the time intervals at each time step.

G Performance measures

We measured the performance of the algorithms using common measures of multi-class classification based on the confusion matrix. A confusion matrix is a specific table layout to visualize the performance of an algorithm. Columns represent the instances in a predicted class and rows represent the instances in an actual class. A straight measure of performance is *accuracy*, defined as the number of correctly classified instances (the sum of the values in the diagonal cells) with respect to their total number. But we are also interested in the analysis of performance at the class/cluster level. Measures defined at class level are *recall*, defined as the fraction of correctly classified instances (*true positives*) with respect to the total number of instances of that class (*true positives* plus *false negatives*), *i.e.*,

$$recall_c = \frac{truePositives}{truePositives + falseNegatives}$$

and *precision*, defined as the fraction of *true positives* with respect to the total number of instances predicted for that class (*true positives* plus *false positives*), *i.e.*,

$$precision_c = \frac{truePositives}{truePositives + falsePositives}$$

As none of these is a reliable measure of performance by itself, it is common to use the *F-measure*, defined as the harmonic mean of *recall* and *precision*, *i.e.*,

$$F_c = \frac{2\, recall_c\, precision_c}{recall_c + precision_c}$$

Finally, a specific measure of performance for multi-class classification is the *macro average F-measure*, defined as the mean of the F-measures of each class, *i.e.*,

$$F = \frac{1}{k}\sum_{c=1}^{k} F_c$$

In contrast to *accuracy*, which is a measure that assigns equal weight to each instance, the *macro average F-measure* assigns equal weight to each class.

H Performance comparison results

We show the performance values of the EMbC, EMC and HMM algorithms on two sets of synthetic trajectories, the first generated using a Markov-chain sampling scheme of the states (Table B), and the second using a mixture-prior sampling scheme (Table C). Values shown correspond to the average macro F-measure and root mean squared error (*i.e.* $F \pm RMSE$) for 100 randomly generated trajectories. The EMC and HMM implementations are highly dependent on a seed value that sets the initial conditions, thus we performed ten different runs for each trajectory. Results denoted as EMC.all and HMM.all correspond to the average of all ten runs over all 100 trajectories and those denoted as EMC.best and HMM.best correspond to the average of the single best run for all trajectories.

The EMC algorithm (as implemented in EMCluster R-package [3, 4]) as well as the HMM algorithm (as implemented in DepmixS4 R-package [5]) both stop, yielding an exception error, when the algorithm lacks convergence. This kind of problems has a strong dependence on a seed value which is randomly chosen at each run. Therefore it is also important to take into account the number of times that each algorithm fails in finding a stable solution. Where specified, the value in parenthesis denotes the percentage of trials that finished yielding a stable solution. It is worth noting that the HMM algorithm fails in some situations, specially when a transition distribution is not present in the data and also when the dataset size is lower.

n	method	$\gamma = 1\%$	$\gamma = 5\%$	$\gamma = 10\%$
50	EMC.all	.5952 ± .1525	.5949 ± .1577	.5996 ± .1550
	EMC.best	.7916 ± .1296	.7796 ± .1268	.7814 ± .1174
	HMM.all	.6939 ± .1508 (53%)	.6917 ± .1602 (55%)	.6958 ± .1649 (52%)
	HMM.best	.8283 ± .1464 (96%)	.8034 ± .1555 (99%)	.8231 ± .1506 (97%)
	EMbC	.7683 ± .1804	.7600 ± .1782	.7771 ± .1711
100	EMC.all	.6479 ± .1518	.6708 ± .1527	.6499 ± .1629
	EMC.best	.8411 ± .1178	.8571 ± .1086	.8565 ± .1295
	HMM.all	.7364 ± .1493 (63%)	.7564 ± .1568 (64%)	.7474 ± .1640 (60%)
	HMM.best	.8781 ± .1217	.9053 ± .1096	.8863 ± .1443
	EMbC	.8438 ± .1265	.8842 ± .0979	.8536 ± .1554
200	EMC.all	.6912 ± .1617	.7111 ± .1625	.7206 ± .1655
	EMC.best	.8766 ± .1088	.9124 ± .0938	.9334 ± .0850
	HMM.all	.7755 ± .1652 (69%)	.7828 ± .1735 (67%)	.7929 ± .1798 (69%)
	HMM.best	.9276 ± .1041	.9450 ± .1060	.9519 ± .1029
	EMbC	.9009 ± .0961	.9263 ± .0797	.9414 ± .0902
400	EMC.all	.7111 ± .1567	.7299 ± .1661	.7555 ± .1712
	EMC.best	.9057 ± .0829	.9341 ± .0874	.9596 ± .0750
	HMM.all	.7706 ± .1784 (77%)	.7992 ± .1756 (78%)	.7889 ± .1794 (79%)
	HMM.best	.9279 ± .1137	.9524 ± .1005 (99%)	.9379 ± .1307
	EMbC	.9028 ± .0849	.9401 ± .0683	.9736 ± .0516
800	EMC.all	.7350 ± .1698	.7601 ± .1763	.7776 ± .1724
	EMC.best	.9172 ± .0813	.9435 ± .0806	.9764 ± .0487
	HMM.all	.7656 ± .1733 (86%)	.7904 ± .1748 (79%)	.7902 ± .1806 (80%)
	HMM.best	.9353 ± .1088	.9649 ± .0841	.9586 ± .1094
	EMbC	.9344 ± .0424	.9559 ± .0537	.9794 ± .0495
1600	EMC.all	.7416 ± .1690	.7822 ± .1735	.7847 ± .1857
	EMC.best	.9207 ± .0852	.9510 ± .0725	.9663 ± .0824
	HMM.all	.7812 ± .1733 (90%)	.7972 ± .1799 (86%)	.7989 ± .1793 (86%)
	HMM.best	.9379 ± .1062	.9548 ± .1034	.9715 ± .0866
	EMbC	.9228 ± .0877	.9651 ± .0437	.9751 ± .0612

Table B: **Performance comparison among EMbC, EMC, and HMM**. Performance with Markov-chain sampled trajectories of different sample sizes n and different definition of the binary regions γ. Values are given as $F \pm RMSE$. Results denoted as EMC.all and HMM.all correspond to the average of all ten runs over all 100 trajectories and those denoted as EMC.best and HMM.best correspond to the average of the single best run for all trajectories. Where specified, the value in parenthesis denote the percentage of trials yielding a stable solution.

n	method	$\gamma = 1\%$	$\gamma = 5\%$	$\gamma = 10\%$
50	EMC.all	.6196 ± .1404	.6277 ± .1516	.6470 ± .1528
	EMC.best	.8107 ± .0952	.8363 ± .0887	.8451 ± .0929
	HMM.all	.6633 ± .1232 (63%)	.6684 ± .1253 (60%)	.6668 ± .1355 (61%)
	HMM.best	.7979 ± .0959	.8026 ± .1108	.8167 ± .1121
	EMbC	.8691 ± .0915	.8837 ± .0900	.8750 ± .0969
100	EMC.all	.6634 ± .1457	.6776 ± .1582	.6814 ± .1615
	EMC.best	.8606 ± .0905	.8828 ± .0891	.8895 ± .1008
	HMM.all	.6635 ± .1360 (69%)	.6751 ± .1273 (70%)	.6719 ± .1317 (71%)
	HMM.best	.8109 ± .1271	.8154 ± .1220	.8255 ± .1394
	EMbC	.8760 ± .0869	.9171 ± .0662	.9174 ± .0882
200	EMC.all	.7229 ± .1613	.7425 ± .1653	.7499 ± .1740
	EMC.best	.9193 ± .0686	.9402 ± .0636	.9639 ± .0615
	HMM.all	.6864 ± .1379 (79%)	.6852 ± .1394 (78%)	.6947 ± .1478 (76%)
	HMM.best	.8544 ± .1226	.8274 ± .1414	.8575 ± .1403
	EMbC	.9180 ± .0654	.9462 ± .0570	.9702 ± .0661
400	EMC.all	.7410 ± .1696	.7713 ± .1681	.7808 ± .1737
	EMC.best	.9375 ± .0494	.9622 ± .0407	.9836 ± .0398
	HMM.all	.7044 ± .1461 (92%)	.6879 ± .1475 (87%)	.6840 ± .1410 (85%)
	HMM.best	.8620 ± .1317	.8842 ± .1367	.8705 ± .1567
	EMbC	.9373 ± .0419	.9587 ± .0414	.9857 ± .0277
800	EMC.all	.7629 ± .1704	.7793 ± .1767	.8006 ± .1753
	EMC.best	.9347 ± .0660	.9711 ± .0443	.9933 ± .0075
	HMM.all	.7020 ± .1490 (99%)	.7246 ± .1585 (97%)	.7179 ± .1554 (94%)
	HMM.best	.8406 ± .1497	.8730 ± .1467	.8832 ± .1568
	EMbC	.9445 ± .0340	.9706 ± .0248	.9920 ± .0179
1600	EMC.all	.7851 ± .1678	.8027 ± .1718	.8045 ± .1780
	EMC.best	.9526 ± .0431	.9812 ± .0104	.9958 ± .0061
	HMM.all	.7202 ± .1533	.7452 ± .1570	.7370 ± .1572 (99%)
	HMM.best	.8658 ± .1476	.8889 ± .1435	.8976 ± .1484
	EMbC	.9520 ± .0317	.9802 ± .0106	.9957 ± .0061

Table C: **Performance comparison among EMbC, EMC, and HMM**. Performance with mixture-prior sampled trajectories of different sample sizes n and different definition of the binary regions γ. Values are given as $F \pm RMSE$. Results denoted as EMC.all and HMM.all correspond to the average of all ten runs over all 100 trajectories and those denoted as EMC.best and HMM.best correspond to the average of the single best run for all trajectories. Where specified, the value in parenthesis denote the percentage of trials yielding a stable solution.

References

[1] Kim J, Min S, Na S, HS C, SH C (2007) Modified GMM training for inexact observation and its application to speaker identification. Speech Sciences 14: 163-175.

[2] Tariquzzaman MD, Kim JY, Na SY, Kim HG (2012) Reliability-Weighted HMM Considering Inexact Observations and its Validation in Speaker Identification. International Journal of Innovative Computing, Information and Control 8.

[3] Chen WC, Maitra R, Melnykov V (2012). EMCluster: EM algorithm for model-based clustering of finite mixture gaussian distribution. R Package, URL http://cran.r-project.org/package=EMCluster.

[4] Chen WC, Maitra R, Melnykov V (2012) A Quick Guid for the EMCluster Package. R Vignette, URL http://cran.r-project.org/package=EMCluster.

[5] Visser I, Speekenbrink M (2010) depmixS4: An R package for hidden markov models. Journal of Statistical Software 36: 1–21.
