# Supplementary material for: Expectation-Maximization Binary Clustering for Behavioural Annotation
Source: PLoS One. 2016 Mar 22;11(3):e0151984. doi: 10.1371/journal.pone.0151984 (PMC4803255; doi:10.1371/journal.pone.0151984)
Supplement: S2 Text — (PDF) [file pone.0151984.s002.pdf]

Generating the Results with the EMbC R-package

J.Garriga; F.Bartumeus
ICREA Movement Ecology Laboratory (CEAB-CSIC)
jgarriga@ceab.csic.es

Version 1.9.3, Dec 2015

Abstract

In this document we give a brief overview on how we have generated the results in the main MS using the EMbC R-package. For detailed information about the package please refer to the package reference manual. The data sets used in this document are available in the supplied S3 data zip file. Please, extract the files in S3Data.zip if you want to run the following chunks of code.

Working Environment

The EMbC package has dependencies with the following packages:

- **methods**, *formal methods and classes* (R Core Team 2015);
- **sp**, *classes and methods for spatial data* (E. J. Pebesma and Bivand 2005; Bivand, Pebesma, and Gomez-Rubio 2013);
- **maptools**, *tools for reading and handling spatial objects* (R. Bivand and Lewin-Koh 2015);
- **mnormt**, *the multivariate normal and t distributions* (Azzalini and Genz 2015);
- **RColorBrewer**, *ColorBrewer palettes* (Neuwirth 2014);

We also suggest the package **rgl**, *3D visualization device system (OpenGL)* (Adler, Murdoch, and others 2015), to allow for dynamic 3D scatter-plots in multivariate analyses.

Also, for researchers who are familiar with the MoveBank framework, we include a special dependence with the package **move**, *Visualizing and analizing animal track data* (Kranstauber and Smolla 2015), to allow users to make use of *Move* objects with its full functionality.

Let's load the package;

```
library(EMbC)
```

```
## Loading required package: move
## Loading required package: sp
## Loading required package: raster
## Loading required package: rgdal
## rgdal: version: 0.8-16, (SVN revision 498)
## Geospatial Data Abstraction Library extensions to R successfully loaded
## Loaded GDAL runtime: GDAL 1.7.3, released 2010/11/10
## Path to GDAL shared files: /usr/share/gdal/1.7
## GDAL does not use iconv for recoding strings.
## Loaded PROJ.4 runtime: Rel. 4.7.1, 23 September 2009, [PJ_VERSION: 470]
## Path to PROJ.4 shared files: (autodetected)
## Loading required package: geosphere
```

Basic structure

Basically, the package consists of a hierarchy of classes with two main components: the *binClst*, the main class, representing the binary clustering of a multivariate data set, and the *binClstPath*, a child class of the former, representing the binary clustering of a trajectory. Each class has its own constructor:

- *embc()*, the main core of the package, implementing the EMbC algorithm itself; this constructor takes as input a matrix of data (with data points given as rows) and returns an object of class *binClst* with the **multivariate** binary clustering of the input data;
- *stbc()*, a specific constructor for the annotation of movement trajectories; this constructor takes as input a data.frame with at least three first columns with *timestamps, longitudes, latitudes*, and returns an object of class *binClstPath* with the **bivariate** (velocity/turn) clustering of the trajectory; eventually it can compute a **trivariate** clustering by including a parameter indicating a solar covariate (either *height* or *azimuth*) to be used as a daytime indicator.

As an example, the package includes an object named *expth*. This is a synthetically generated trajectory stored as a data.frame with three columns with *time-stamps*, *longitudes* and *latitudes*. To perform the bivariate velocity/turn clustering of this trajectory we simply call the *stbc()* constructor passing in the path data.frame and naming the output *binClstPath* object (*e.g. mybc*);

```
mybc <- stbc(expth)
```

```
## ... computing starting delimiters:
## ... delimiter  lL :  1.488106
## ... delimiter  lH :  1.488106
## ... delimiter  hL :  1.488106
## ... delimiter  hH :  1.488106
## ... delimiter  Ll :  1.982173
## ... delimiter  Lh :  1.982173
## ... delimiter  Hl :  0.8682456
## ... delimiter  Hh :  0.8682456
## [1]   0  -0.0000e+00       4       600
## [1]   1  -4.2724e+00       4       465
## [1]   2  -4.1181e+00       4        35
## [1]   3  -3.6766e+00       4        34
## [1]   4  -3.2744e+00       4        24
## [1]   5  -3.1224e+00       4        21
## [1]   6  -3.0675e+00       4        15
## [1]   7  -3.0520e+00       4         7
## [1]   8  -3.0456e+00       4         4
## [1]   9  -3.0416e+00       4         0
## [1]  10  -3.0395e+00       4         0
## [1]  11  -3.0384e+00       4         0
## [1]  12  -3.0379e+00       4         0
## [1]  13  -3.0377e+00       4         0
## [1]  14  -3.0376e+00       4         0
## [1] ... Stable clustering
```

At each step, the algorithm shows the current iteration number, the likelihood value, the number of effective clusters and the number of data points that have changed their labels with respect to the previous iteration.

The behaviour of the constructors can be modified by means of different parameters (*e.g.* control the level of information shown at each step of the algorithm, perform a pre-smoothing of the data).

The *binClst* and *binClstPath* objects include several slots through which we can have acces to all the information related to the clustering (input data, intermediate computations and output data).

```
slotNames(mybc)
```

```
##  [1] "pth"       "spn"       "dst"       "hdg"       "bursted"   "tracks"
##  [7] "midPoints" "X"         "U"         "stdv"      "m"         "k"
## [13] "n"         "R"         "P"         "W"         "A"         "L"
## [19] "C"
```

The most basic slots of a *binClstPath* object are:

- mybc@n, the number of data points (*i.e.* the length of the path);
- mybc@m, the number of input features;
- mybc@k, the initial (maximum) number of clusters, given by 2^m;
- mybc@pth, the input data.frame with three first columns named as *dTm*, *lon*, *lat*;
- mybc@spn, a numeric vector with the time span between locations;
- mybc@dst, a numeric vector with the estimated distances between locations, computed as loxodromic lines;
- mybc@hdg, a numeric vector with the heading directions at each location;
- mybc@X, a matrix with the input data points, (estimated local values of velocity and turn);
- mybc@U, a matrix (of equal dimension as mybcp@X) with a reliability value associated to each data value;
- mybc@R, a matrix with the values of the delimiters for each binary region;
- mybc@P, a list with an element for each cluster, where each element is a named list with the Gaussian parameters of the corresponding Gaussian component;
- mybc@A, a numeric vector with the output labelling of each location, (coded as 1:LL, 2:LH, 3:HL, and 4:HH)
- mybc@W, a $(n \times k)$ matrix with the likelihood of each location of belonging to each one of the clusters (column ordered);
- mybc@C, a numeric vector with color codes to distinguish the clusters in the plots.

All slots are accessible and can be used with any R function external to the package or even modified. However, **we recommend not to manually change the values in the slots in order to keep the internal consistency**.

The package includes a set of complementary functions to easily visualize the output and make further analyses.

Data Description and Exploratory Analysis

In the following we describe the empirical data sets used in the main text and we spell out how we generated the results using the EMbC R-package.

Cory's shearwater (*Calonectris diomedea*) foraging trajectory

This is a high resolution GPS trajectory with GNSS readings scheduled at intervals of 3 minutes. The track spans for 4 days, starting at 2012-08-18 19:43:22 and ending at 2012-08-23 18:25:17, with a total of 2543 fixes and a mean time interval of 155 seconds.

Let's load the data set. A common source of problems is that time stamps are not correctly supplied as POSIXct, (therefore the subsequent transformation of time stamps);

```
shw <- read.csv('shw_dta.csv',header=TRUE)
shw$tStamp <- as.POSIXct(strptime(shw$tStamp,'%F %X'))
```

```
head(shw)
```

```
##   id              tStamp      lon      lat
## 1  0 2012-08-18 19:43:22 3.871870 40.06417
## 2  1 2012-08-18 19:46:02 3.872594 40.06423
## 3  2 2012-08-18 19:48:41 3.876514 40.06645
## 4  3 2012-08-18 19:51:21 3.876545 40.06572
## 5  4 2012-08-18 19:54:00 3.875511 40.06385
## 6  5 2012-08-18 19:56:39 3.875824 40.06142
```

To perform the standard velocity/turn clustering of the trajectory, we just call the constructor by passing a data.frame with the trajectory (note that the data.frame includes a first column with a row *id* that we are discarding to meet the required format);

```
# info=-1 suppresses any iteration step information
shwbc <- stbc(shw[,2:4],info=-1)
```

```
## [1]   0  -0.0000e+00       4      2543
## [1] ... Stable clustering
```

We can plot the step likelihood to check convergence, (we can set an iteration offset for a better visualisation of the last part of the optimization process);

```
lkhp(shwbc)         # left panel
lkhp(shwbc,25)      # right panel
```

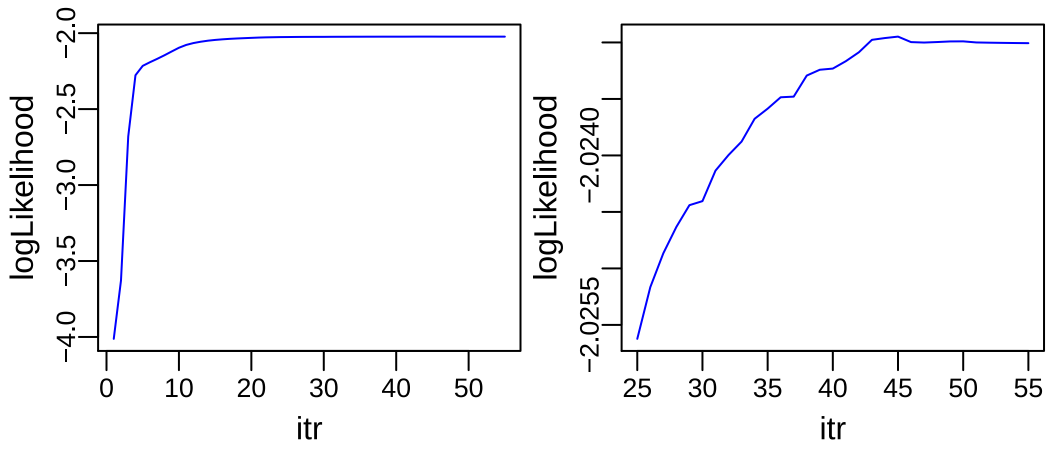

The set of parameters of the Gaussian mixture is accessible through the slot shwbc@P. The function *stts()* offers a fast compact view of the parameter set. For each cluster we have two columns per feature (*i.e.* velocity and turn) with the mean and standard deviation of the corresponding Gaussian component. The last two columns show the marginal distribution of the cluster in absolut and percentage values;

```
stts(shwbc)
```

```
##           X1.mn    X1.sd    X2.mn    X2.sd     kn  kn(%)
##   1 LL     0.43     0.32     0.18     0.15    961  37.79
##   2 LH     0.21     0.12     1.57     0.89    873  34.33
##   3 HL     8.79     2.45     0.12     0.10    327  12.86
##   4 HH     3.75     3.20     1.56     0.89    381  14.98
```

The shwbc@R slot is a matrix with the values that delimit each binary region. For each cluster we have the min and max values of each variable (*i.e.* velocity and turn);

```
shwbc@R
```

```
##         X1.min    X2.min     X1.max    X2.max
## 1.LL 0.0000000 0.0000000  1.6433807 0.5148351
## 2.LH 0.0000000 0.5148351  0.5732197 3.1415921
## 3.HL 1.6433807 0.0000000 13.6262610 0.3743097
## 4.HH 0.5732197 0.3743097 13.6262610 3.1415921
```

We can see the clustering as a coloured scatter-plot of the input data with grey lines depicting the values of the delimiters;

```
# NC in the legend stands for not classified data points,
# (e.g. the last point of the trajectory)
sctr(shwbc)
```

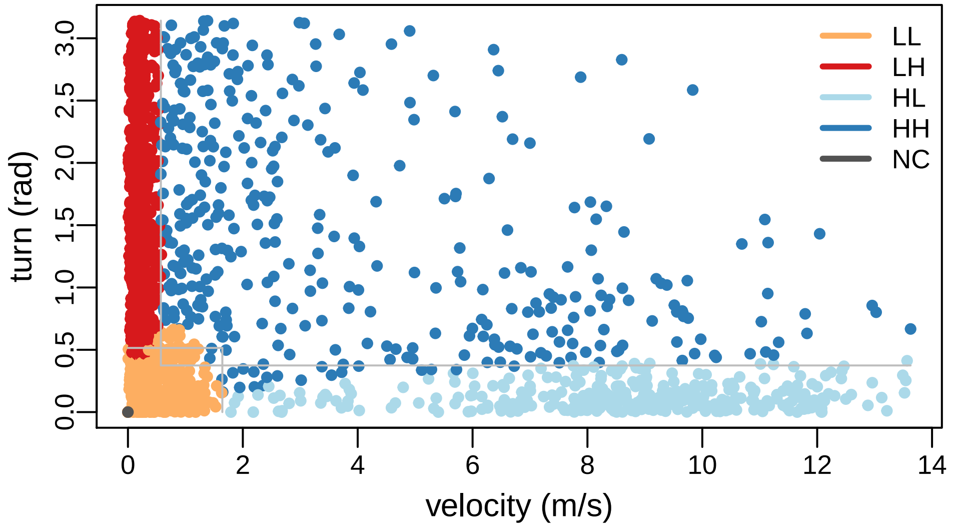

We can make use of the labelling values stored in shwbc@A and the values of the delimiters stored in shwbc@R to visualize the binary splitting of velocities separately for low and high values of turns;

```
hist(shwbc@X[which(shwbc@A%in%c(1,3)),1],breaks=seq(0,max(shwbc@X[,1]),
    max(shwbc@X[,1])/50),include.lowest=TRUE,xlim=range(shwbc@X[,1]),
    xlab='velocity (m/s)',main='velocity distribution for LOW turns',cex.main=0.8)
abline(v=shwbc@R[1,3],col=1,lwd=1.5,lty='dashed')
hist(shwbc@X[which(shwbc@A%in%c(2,4)),1],breaks=seq(0,max(shwbc@X[,1]),
    max(shwbc@X[,1])/50),include.lowest=TRUE,xlim=range(shwbc@X[,1]),
    xlab='velocity (m/s)',main='velocity distribution for HIGH turns',cex.main=0.8)
abline(v=shwbc@R[4,2],col=1,lwd=1.5,lty='dotdash')
```

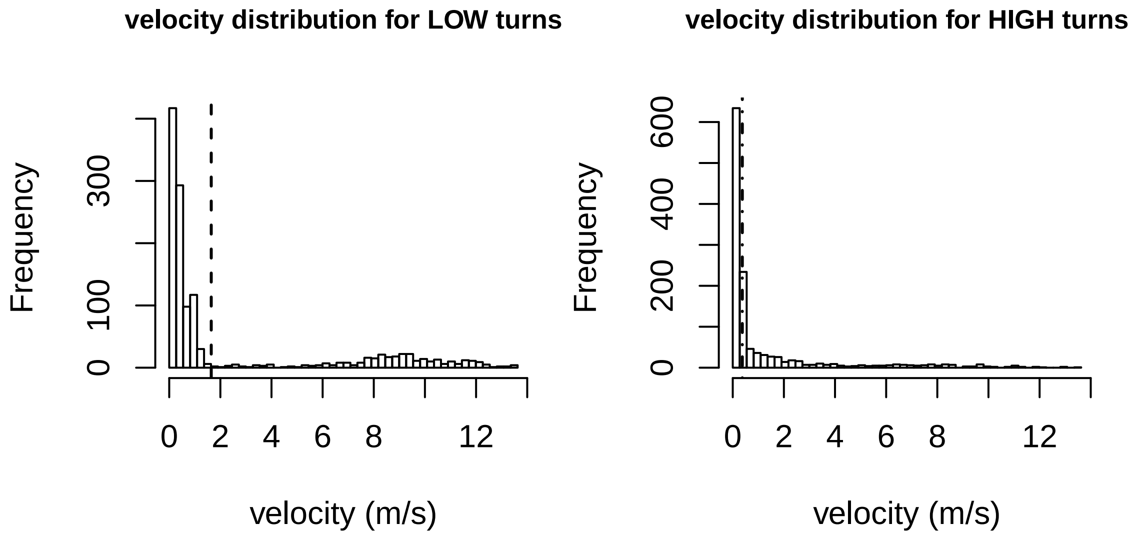

Or, we can see the binary split of turns separately for low and high values of velocity;

```
hist(shwbc@X[which(shwbc@A%in%c(1,2)),2],breaks=seq(0,pi,pi/50),
    xlab='turn (rad)',main='turn distribution for LOW velocities',cex.main=0.8)
abline(v=shwbc@R[1,4],col=1,lwd=1.5,lty='dashed')
hist(shwbc@X[which(shwbc@A%in%c(3,4)),2],breaks=seq(0,pi,pi/50),
    xlab='turn (rad)',main='turn distribution for HIGH velocities',cex.main=0.8)
abline(v=shwbc@R[3,4],col=1,lwd=1.5,lty='dotdash')
```

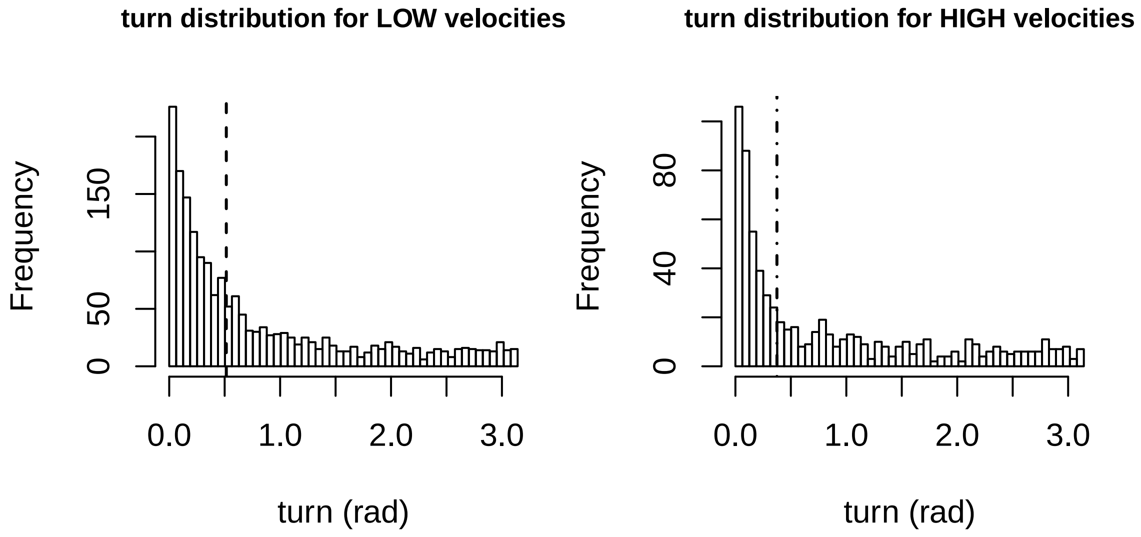

We can perform a fast visual check of the behaviourally annotated trajectory. Each location is depicted with the colour of the cluster it belongs to. Also, a behavioural temporal profile is included;

```
# the parameter lims allows focussing on a particular portion of the trajectory
view(shwbc,lims=c(280,430))
```

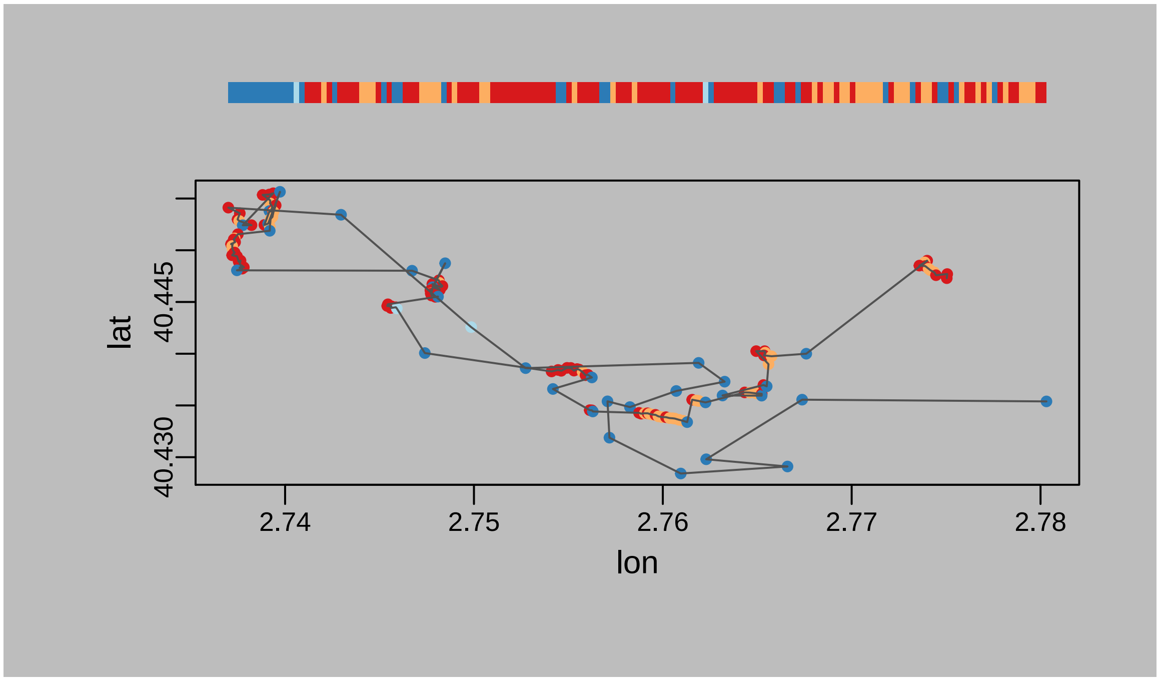

Osprey (*Pandion haliateus*) migration trajectory

This is an Argos trajectory with the positioning system scheduled at intervals of 1 hour between 03.00h and 20.00h. The track spans from 2006-08-29 06:00:00 to 2006-10-29 20:00:00, with a total of 594 fixes and a mean time interval of 3600 seconds.

Let's load the data set;

```
osp <- read.csv('osp_dta.csv',header=TRUE)
osp$tStamp <- as.POSIXct(strptime(osp$tStamp,'%F %X'))
```

```
head(osp)
```

```
##   id              tStamp      lon      lat
## 1  0 2006-08-29 06:00:00 15.47183 59.61883
## 2  1 2006-08-29 07:00:00 15.47600 59.62467
## 3  2 2006-08-29 08:00:00 15.55983 59.57050
## 4  3 2006-08-29 15:00:00 15.60250 59.58933
## 5  4 2006-08-29 16:00:00 15.60150 59.58850
## 6  5 2006-08-29 17:00:00 15.60083 59.58783
```

Now, let's perform the standard velocity/turn clustering of the trajectory;

```
ospbc0 <- stbc(osp[,2:4],info=-1)
```

```
## [1]   0  -0.0000e+00       4       594
## [1] ... Stable clustering
```

For comparative purposes, it might be interesting to perform a velocity/turn clustering of the trajectory with a pre-smoothing of 6 hours. In this case, the constructor will estimate local values of velocity and turn averaged within a time window centered at each location and spanning for the time interval specified with the parameter *smth* (in hours);

```
ospbc6 <- stbc(osp[,2:4],smth=6,info=-1)
```

```
## [1]   0  -0.0000e+00       4       594
## [1] ... Stable clustering
```

The function *lblp()* depicts a two panel figure with the temporal series of the input features along with the corresponding temporal behavioural profile;

```
lblp(ospbc6)
```

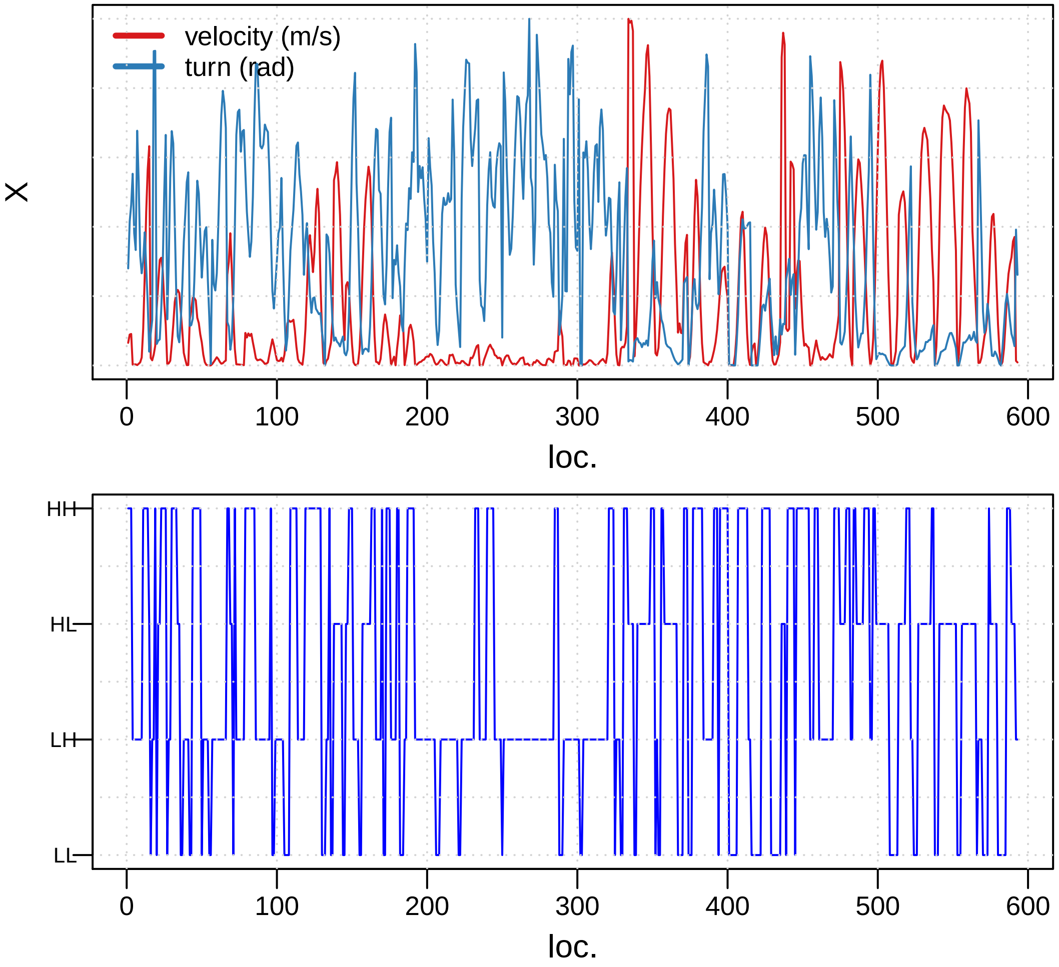

Regardless of the pre-smoothing, we can still apply a post-smoothing. The *smth()* function returns a copy of the input *binClstPath* with a smoothed output labelling;

```
ospbc6smoothed <- smth(ospbc6)
```

The function *cnfm()* computes the confusion matrix of a *binClstPath* object with respect to another one or with respect to a reference (expert) labelling. For instance, we can numerically asses the effect of applying a full (pre/post) smoothing with respect to the standard non-smoothed clustering;

```
# Note that we can pass the post-smoothed clustering without instantiating it
# (useful in case of large trajectories to optimize memory usage)
cnfm(smth(ospbc6),ospbc0)
```

```
##      cls.01 cls.02 cls.03 cls.04   mrg.   Prc.
## 1.LL     63      7     26      8   0.18   0.61
## 2.LH     29    159      8     20   0.36   0.74
## 3.HL      5      1     99      3   0.18   0.92
## 4.HH      7     41     45     72   0.28   0.44
##      ------ ------ ------ ------ ------ ------
## mrg    0.18   0.35   0.30   0.17   0.66   0.68
## Rcl    0.61   0.76   0.56   0.70   0.66    NaN
## Fms    0.61   0.75   0.70   0.54    NaN   0.65
```

As a consequence of the smoothing, 26 cls.03 (HL) isolated relocation data points in *ospbc0* are rellabeled as LL, hence absorbed into their framing resting behaviour. Conversly, 5 cls.01 (LL) resting locations in *ospbc0*, corresponding to isolated stops framed in a relocation context are rellabeled as HL, therefore emphasizing the fly-ways. In general, smoothing will emphasize the dominant behaviours at each part of the trajectory.

We can make a visual check of the output versus some validation feature (*e.g.* environmental information). The function *chkp()* depicts the temporal labelling profile versus the validation feature shown as coloured background bars. By default, day/night time information is used for validation;

```
# the parameter lims allows focusing on a portion of the trajectory
# (useful for long trajectories)
chkp(smth(ospbc6),lims=c(200,ospbc6@n))
```

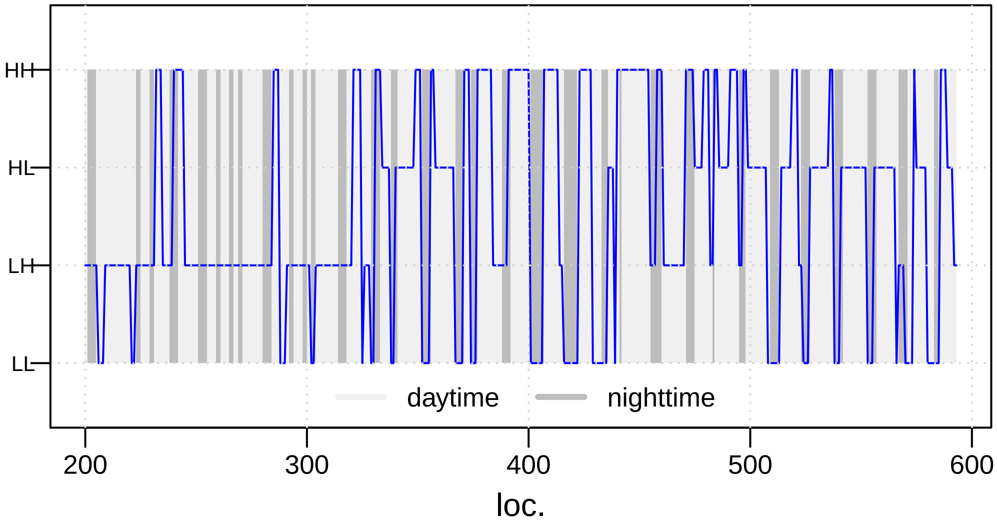

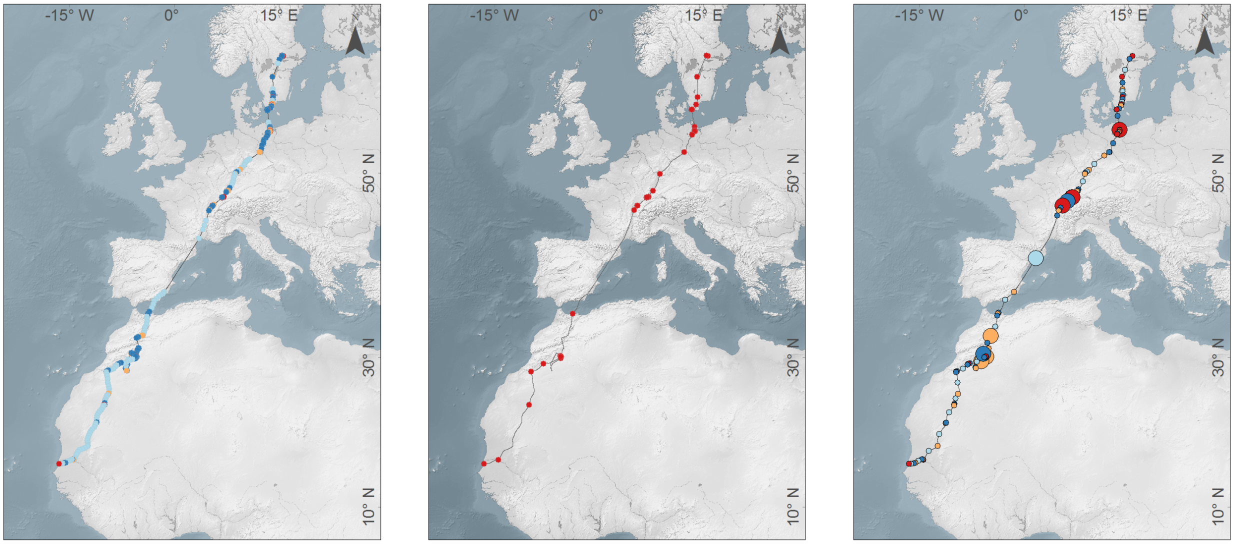

Table 1: Osprey (*Pandion haliateus*) migration trajectory. Different kml views of the annotated trajectory; left: point-wise view; centre: point-wise view showing only the LH locations (stopovers); right: burst-wise view of the whole trajectory. Due to copyright restrictions the figure is for representative purposes only. Source: Made with Natural Earth; Free vector and raster map data @ naturalearthdata.com.

For a detailed inspection of the annotated trajectory we can generate a kml doc and use the Google-earth application to examine it. The kml documents can be generated either point-wise (location-wise) or burst-wise (*i.e.* using the proposed bursted visualisation);

```
# generate a point-wise kml doc
pkml(ospbc6)
# generate a point-wise kml doc showing only the stopover areas (HL cluster)
pkml(ospbc6,showClst=c(2))
# generate a burst-wise kml doc and launch Google-earth within R
bkml(ospbc6,markerRadius=25,display=TRUE)
# warning: depending on the system's configuration, display=TRUE can return an error
# message after closing Google-earth but this error does not affect R
```

By default the *pkml()* and *bkml()* functions save the kml file with a *Sys.time()* based name in the *embcDocs* folder in the user's home directory (creating it if it does not exist), but this default behaviour can be changed. In Table 1 we show some figures taken from the generated kml documents.

Straw-coloured fruit bat (*Eidolon helvum*) roosting/foraging trajectory

This is a GPS trajectory with GNSS readings scheduled at intervals of 5 minutes between 00.00h and 23.00h. The track spans from 2011-02-03 17:59:23 to 2011-02-09 06:00:00, with a total of 434 fixes and a mean time interval of 299 seconds.

Let's load the data set;

```
bat <- read.table('bat_dta.csv',sep='\t',header=TRUE)
bat$tStamp <- as.POSIXct(strptime(bat$tStamp,'%F %X'))
```

```r
head(bat)
```

```
##    id              tStamp        lon      lat explbl explbl2
## 1 718 2011-02-03 17:59:23 -0.1991556 5.623138      3 commute
## 2 724 2011-02-03 18:05:00 -0.2111885 5.641334      3 commute
## 3 730 2011-02-03 18:10:00 -0.2261752 5.656798      3 commute
## 4 736 2011-02-03 18:15:01 -0.2384989 5.672893      3 commute
## 5 742 2011-02-03 18:19:59 -0.2442364 5.692220      3 commute
## 6 748 2011-02-03 18:25:01 -0.2579482 5.717943      3 commute
```

In this case we perform a velocity/turn clustering of the trajectory with a subsequent smoothing of the output. Note that we include a fourth column with expert labelling. There is no problem in doing so as long as the first three columns correspond to time-stamp, longitude and latitude as required;

```r
batbc <- smth(stbc(bat[,2:5],info=-1))
```

```
## [1]   0  -0.0000e+00       4       434
## [1] ... Stable clustering
```

```r
stts(batbc)
```

```
##          X1.mn    X1.sd    X2.mn    X2.sd     kn  kn(%)
##   1 LL    2.26     5.27     0.18     0.20     27   6.22
##   3 HL   12.07     2.57     0.08     0.09    204  47.00
##   4 HH    0.75     1.53     1.91     0.87    202  46.54
```

When used with two arguments, the *lblp()* function allows us to compare the temporal labelling profile of a *binClstPath* object with respect to another one, or with respect to a reference (expert) labelling. In this case, we use it to compare the post-smoothed version with a non-smoothed one (not instantiated).

```r
lblp(stbc(bat[,2:5],info=-1),batbc)
```

```
## [1]   0  -0.0000e+00       4       434
## [1] ... Stable clustering
```

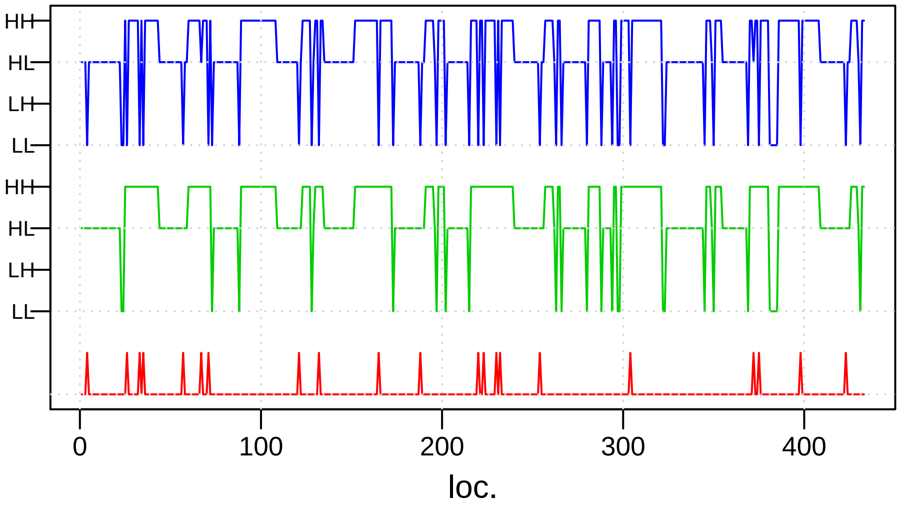

For this data set, we get a clustering with a very special lay-out, with some interesting particularities. Let's see the scatter-plot;

```
sctr(batbc)
```

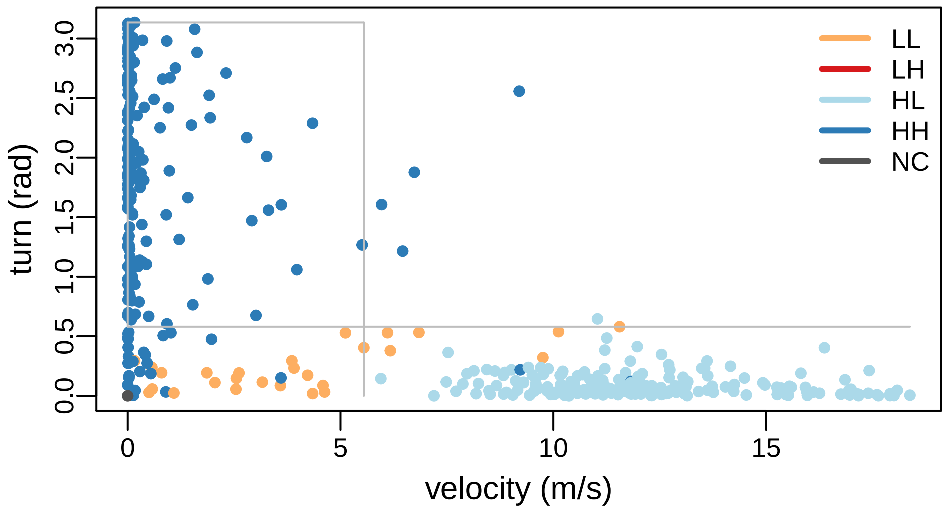

First, we can see how the post-smoothing has changed the label of some data points that clearly appear out of their binary region;

Second, let's note that clusters LH and HH have been merged into a single one. The resulting cluster has been labelled as HH but it could have been labelled LH as well. When merging occurs, the semantics of the final labelling is somewhat misleading because the final labelling is only a result of how the algorithm evolved until reaching the merging point. In any case, the label should be read as XH, that is, by taking into account that the first feature (in this case the velocity) is meaningless given the values of the rest (*i.e* velocities can be either H or L given H values of turn). This situation is more likely to occur as the number of dimensions increases.

The package does not deal with labels of the type XH. However, one can change labels as desired (even to manually force the merging of two clusters). In this case, and given the position of the HH cluster, we feel more comfortable by relabelling the cluster HH (cluster number 4) as LH (cluster number 2);

```
rlbl(batbc,4,2)
sctr(batbc)
```

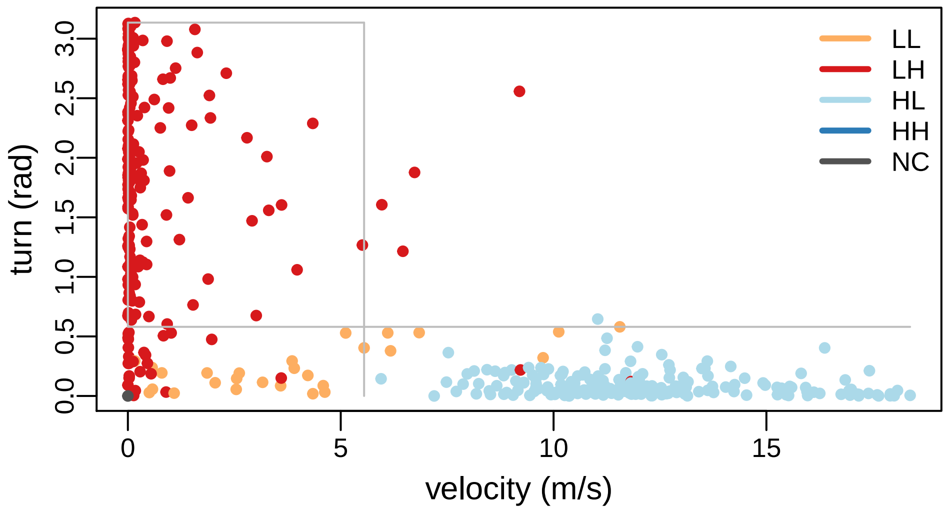

It is important to note that relabelling is done in the given instance instead of returning a relabelled copy of it, but the likelihood weights, the Gaussian parameters and the delimiters are not recomputed. The advantage is that the labelling can be reset to its original situation (please, refer to the package reference manual).

Third, let's note that the LL cluster spreads along the *X1* axis suggesting a semantics that clearly differ from the standard semantics (*i.e.* resting behaviour) of a LL velocity/turn cluster. This is so because the GPS was turned off during the day and fixes occured only when the animal moved during the night, hence this data set represents a strongly biased sampling of the animal's behaviour, the resting behaviour being completely removed. Let's have a look to the temporal behavioural profile;

```
chkp(batbc)
```

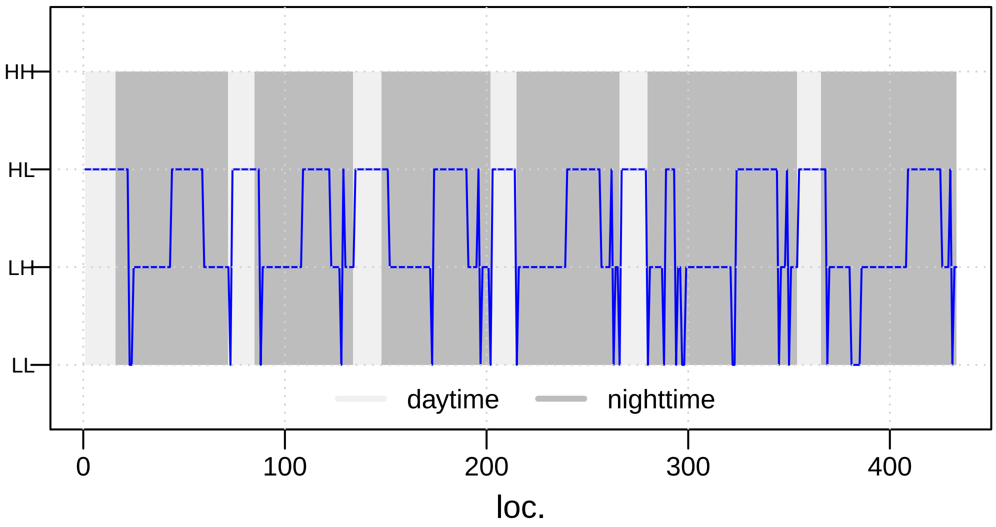

We observe a quite regular pattern in which some of the LL locations appear systematically as a transient state between LH (foraging) and HL (resting) periods. Also, by examining a kml document we see that some of the resting LL locations correspond to the same place in the daily relocation of the animal. In Table 2 we show a view of the kml docs generated with the code below.

```
# generate a point-wise kml doc
pkml(batbc)
# generate a point-wise kml doc showing only the LL locations
pkml(batbc,showClst=c(1))
```

In summary, instead of a resting behaviour, the LL cluster represents a different behaviour probably related with the use of landmarks as references in the daily relocations of the animal.

Finally, as we have an expert labelling available we can asses the degree to which the EMbC labelling captures what an expert might be looking for (note that the expert is likely to be using information that is not really conveyed by our input features). Numerical assesments of this kind are done by means of confusion matrices, hence the number of expert classes should match, or not be greater than, the number of classes (see the package reference manual). This is straightforward as long as a column named *lbl* is included with the input data.frame (we did so but with a different column name);

```
# let's first relabel the column with the expert labelling
colnames(batbc@pth)[4] <- 'lbl'
```

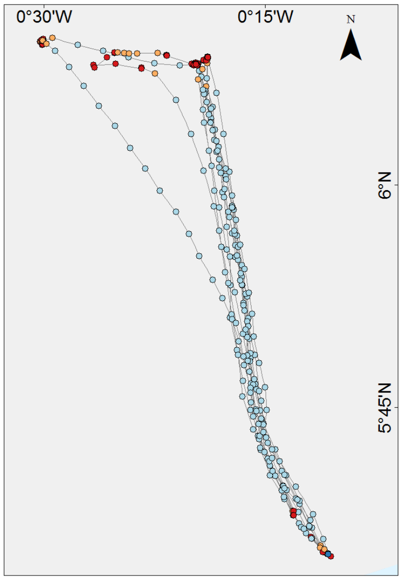

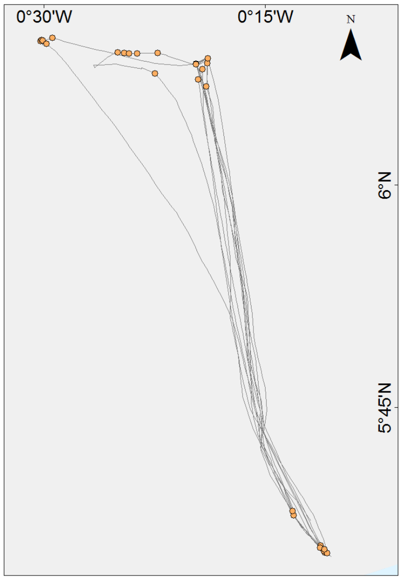

Table 2: Straw-coloured fruit bat (*Eidolon helvum*) foraging trajectory. Point-wise kml views of the annotated trajectory; left: all locations are shown; right: only locations of the LL cluster are shown. Due to copyright restrictions the figure is for representative purposes only. Source: Made with Natural Earth; Free vector and raster map data @ naturalearthdata.com.

```
# expert labelling: cls.01:roost, cls.02:forage, cls.03:commute
cnfm(batbc)
```

```
##       cls.01 cls.02 cls.03 cls.04   mrg.   Prc.
## 1.LL       2     12     13      0   0.06   0.07
## 2.LH      21    175      6      0   0.47   0.87
## 3.HL       0      0    204      0   0.47   1.00
## 4.HH       0      0      0      0   0.00   0.00
##       ------ ------ ------ ------ ------ ------
## mrg     0.05   0.43   0.52      0   0.88   0.48
## Rcl     0.09   0.94   0.91      0   0.48    NaN
## Fms     0.08   0.90   0.95      0    NaN   0.48
```

References

Adler, Daniel, Duncan Murdoch, and others. 2015. *Rgl: 3D Visualization Using OpenGL*. http://CRAN.R-project.org/package=rgl.

Azzalini, Adelchi, and Alan Genz. 2015. *The R Package `mnormt`: The Multivariate Normal and t Distributions (Version 1.5-3)*. http://azzalini.stat.unipd.it/SW/Pkg-mnormt.

Bivand, Roger S., Edzer Pebesma, and Virgilio Gomez-Rubio. 2013. *Applied Spatial Data Analysis with R, Second Edition*. Springer, NY. http://www.asdar-book.org/.

Bivand, Roger, and Nicholas Lewin-Koh. 2015. *Maptools: Tools for Reading and Handling Spatial Objects*. http://CRAN.R-project.org/package=maptools.

Kranstauber, Bart, and Marco Smolla. 2015. *Move: Visualizing and Analyzing Animal Track Data*. http://CRAN.R-project.org/package=move.

Neuwirth, Erich. 2014. *RColorBrewer: ColorBrewer Palettes*. http://CRAN.R-project.org/package=RColorBrewer.

Pebesma, Edzer J., and Roger S. Bivand. 2005. "Classes and Methods for Spatial Data in R." *R News* 5 (2): 9–13. http://CRAN.R-project.org/doc/Rnews/.

R Core Team. 2015. *R: A Language and Environment for Statistical Computing*. Vienna, Austria: R Foundation for Statistical Computing. http://www.R-project.org/.
